# Transcription factor NFκB regulates the expression of the histone deacetylase SIRT1

**DOI:** 10.1186/1868-7083-5-11

**Published:** 2013-07-19

**Authors:** Judith Katto, Nicole Engel, Wasim Abbas, Georges Herbein, Ulrich Mahlknecht

**Affiliations:** 1 José Carreras Center for Immunotherapy and Gene Therapy, Saarland University Medical Center, Kirrbergerstraße Bldg. 45.3, 66421, Homburg/Saar, Germany; 2 Department of Internal Medicine, University of Heidelberg Medical Center, Im Neuenheimer Feld 410, 69120, Heidelberg, Germany; 3 Department of Virology, Pathogens & Inflammation Research Unit UPRES EA4266, SFR FED 4234, University of Franche-Comté, CHU Besançon, Besançon, France; 4 Department of Haematology/Oncology, St. Lukas Klinik Solingen, Schwanenstraße 132, 42697, Solingen, Germany

**Keywords:** Protein deacetylase SIRT1, Nuclear factor NF-kappa-B, Apoptosis

## Abstract

**Background:**

The NAD-dependent protein deacetylase SIRT1 has a wide range of different targets, which may be regulated either directly through deacetylation and thus potentially altering their activity or localization or indirectly by deacetylation of histones, which in turn alters their transcription rate and availability. SIRT1 is therefore involved in the regulation of many different and fundamental cellular processes such as apoptosis, metabolism, differentiation and cell cycle arrest. It is also involved in the regulation of resistance of cells against oxidative stress and longevity under conditions of caloric restriction. Even though the targets and role of SIRT1 have been studied quite intensively, only little is known about the mechanisms affecting *SIRT1* transcriptional regulation. The nuclear factor NFκB is a well-studied and widely known transcription factor, which is involved in the regulation of many important cellular activities. The regulation of NFκB by SIRT1 has been reported recently, but it is, however, still unknown whether a feedback mechanism affects the regulation of *SIRT1* too, particularly in view of the fact that putative NFκB binding sites within the *SIRT1* promoter suggest just that.

**Results:**

In the study presented herein we show that there is activation of the *SIRT1* promoter by overexpression of different NFκB subunits. Direct binding of NFκB to the *SIRT1* promoter can be demonstrated by an electrophoretic mobility shift assay. Further investigations indicated enhanced expression of *SIRT1* on the mRNA levels in cells overexpressing NFκB. A functional assay showed that acetylation of one of the main target proteins of SIRT1 is reduced in these cells.

**Conclusions:**

These finding together indicate SIRT1 expression to be regulated in a positive feedback loop by NFκB. The putative binding sites for NFκB found within the *SIRT1* promoter appears to be functional and several NFκB subunits are able to enhance the expression of SIRT1 if they are overexpressed.

## Background

The regulation of activity, cellular localization and stability of nearly all proteins depends on post-transcriptional and/or post-translational modifications. Such modifications are being carried out by a diversity of chemical reactions including (de)phosphorylation, (de)acetylation or ubiquitination and many others. These highly dynamic regulatory processes are controlled and driven by antagonizing enzymes and allow cells to react to environmental changes and to communicate with each other. Histone deacetylases (HDACs) are one such group of enzymes. HDACs are ubiquitous and catalyze the removal of acetyl groups from both histones and non-histone proteins. The most obvious and best examined role of HDACs is their effect on the constitution and maintenance of chromosomal structure as well as the regulation of global gene expression through modification of the so called histone code and alteration of the accessibility of genes for transcription factors [1]. However, apart from their role as superordinative gene regulators and their role in the control of chromatin structure, some proteins of this family are associated with the distinct regulation of single target proteins, which may specifically be deacetylated [2].

Histone deacetylases are divided into four different classes: three groups of classical HDACs known as class I, class II and class IV HDACs and a fourth class, also being referred to as the ‘sirtuins’ [3]. The sirtuins differ from the other classes of HDACs based on their dependence on the presence of the coenzyme NAD^+^[4]. Seven human sirtuins, which are homologs of the yeast silent information regulator 2 (SIR2) protein, have been reported so far. SIR2 is responsible for the transcriptional silencing of silent mating loci, telomeres and ribosomal DNA in yeast. It also suppresses the recombination of rDNA and thereby lowers the formation of toxic extrachromosomal rDNA rings, which extends lifespan in yeast cells dramatically [5]. Yeast SIR2 or mammalian sirtuin overexpression goes along with the global deacetylation of histones [6]. Most of the gene silencing effects that are being associated with sirtuin activity depend on the deacetylation of histone H4 lysine 16 (H4_K16_) [7] and/or histone H3 lysines 9, 14 or 18 (H3_K9_, H3_K14_, H3_K18_) [8]. Depending on the presence of NAD^+^, the sirtuins are biological sensors for the cellular energetic and oxidative status. Thus, high levels of NAD^+^ go along with high sirtuins activity [9].

The human deacetylase sirtuin1 (SIRT1) shares the highest degree of homology of all mammalian sirtuins with the yeast protein SIR2. SIRT1 localizes predominantly to the cell nucleus [10] and deacetylates both histones (preferably H4_K16_) and non-histone proteins. SIRT1 antagonizes the histone acetyltransferase p300 [11], which acetylates all histones and some non-histone proteins such as p53 and FOXO3a. SIRT1 is involved in a number of cellular regulation mechanisms affecting cellular lifespan, stress resistance, apoptosis and cell metabolism [12]. The prolongation of cellular lifespan under conditions of caloric restriction has been known for quite some time. It has been described for a number of very different organisms ranging from yeast [13] to nematodes [14] to mammals [15,16] and has been reported to be associated with SIRT1. Caloric restriction causes an increase in the production of NAD^+^ and goes along with increased levels of *SIRT1* mRNA [13,16] as well as with increased SIRT1 activity [17]. The positive influence of SIRT1 on longevity in mammals has been reported to depend on increased stress resistance and reduced apoptosis. In addition to the modification of histone proteins, SIRT1 also carries the ability to inactivate a number of non-histone proteins by deacetylation. One of the most prominent examples for this mechanism is the deacetylation of tumor suppressor protein p53. The transcription factor p53 triggers apoptosis in the case of DNA damage or oxidative stress and plays a key role in cellular defense against the development of malignant transformation [18]. The inactivation of p53 by SIRT1 may retard apoptosis and render cells more resistant to oxidative stress [19,20]. On the other hand, overexpression of SIRT1 may also promote carcinogenesis because of its inhibitory effect on apoptosis and cell cycle arrest. The forkhead proteins constitute another group of proteins that is also involved in the regulation of apoptosis, stress resistance and metabolism; these proteins are reported to be regulated by SIRT1 [21].

Another protein that may be deacetylated by SIRT1 is the nuclear factor NF-kappa-B (NFκB) [22-24]. NFκB is a ubiquitously distributed transcription factor that controls gene expression of many target genes, thereby affecting important cellular functions such as cell cycle, angiogenesis, adhesion and apoptosis [25]. NFκB is being constituted by homo- and heterodimers of different NFκB/Rel-proteins, which all share a highly conserved DNA binding/dimerization domain called ‘Rel homology (RH) domain’ [26]. NFκB/Rel proteins can be divided into two classes. Members of the first class are coded by the genes *NFKB1* and *NFKB2* both of them normally being transcribed as long isoforms called NFκB-p105 and NFκB-p100, which are inactive as long as they have inhibiting C-terminal domains. The shorter active forms of this class, the NFκB-p50 (derived from p105) and NFκB-p52 (derived from p100) are produced by arrested translation or partial proteolysis of the longer forms. The subunit NFκB-p49 is another isoform that can be generated from an alternatively spliced transcript of p100. It is reported to be involved in the activation of the HIV enhancer [27]. The second class contains the proteins c-Rel, RelA (also called NFκB-p65) and RelB, which may activate gene expression in homodimers as well as in heterodimers [28]. The most prevalent and best studied dimer is the heterodimer consisting of the p65 and p50 protein. NFκB is constitutively transcribed in many different cell types, but it remains inactive because the dimers are bound to inhibitory proteins of the IκB family, which mask their nuclear translocation signal and consequently go along with their localization in the cell cytoplasm. In response to diverse stimuli IκB proteins are phosphorylated by IκB kinase (IKK) which triggers their ubiquitination and thus their degradation by the proteasome machinery. NFκB is subsequently translocated to the nucleus were it interacts with promoters, thus activating the expression of specific target genes [29].

Generally speaking, alterations of *SIRT1* gene expression are associated with changes in cell metabolism, differentiation, proliferation and many other fundamental cell features and may therefore affect numerous diseases such as diabetes, cancer or arteriosclerosis. Therefore, regulation of *SIRT1* gene expression at the promoter level is of great interest in order to further elucidate our understanding of the molecular mechanisms that involve SIRT1 in both health and disease. The goal of the study presented herein consists in the further elucidation of the interplay of the transcription factor NFκB, in the reciprocal regulation with SIRT1, taking into consideration possible feedback mechanisms in the regulation of these two proteins.

## Results and discussion

### Human *SIRT1* gene promoter analysis

Alterations in the SIRT1 expression rate are associated with changes in cell metabolism, differentiation, proliferation and many other fundamental cell features and affect many diseases such as diabetes, cancer or arteriosclerosis. But at present little information is available on the molecular mechanisms regulating *SIRT1* at the promoter level. In order to further elucidate the regulation of *SIRT1* at the promoter level, a search for putative transcription factor binding sites was carried out using the online tool MOTIF Search (http://motif.genome.jp). The search was based on the alignment with the transcription factor database TRANSFAC. Among others, we found the *SIRT1* promoter to contain several putative NFκB binding sites. NFκB is a transcription factor that is involved in apoptosis, the regulation of cellular stress, and numerous immunological functions. The NFκB consensus sequence GGGAMTTYCC with M and Y being exchangeable bases was found with minor variations in three positions of the *SIRT1* promoter. The three mentioned positions are located at the promoter base-pair positions 1,168 to 1,177 bp, 1,441 to 1,450 bp and 1,838 to 1,847 bp. In accordance with our *in silico* analyses the latter two NFκB potential binding sites appeared to be specific for the NFκB subunit p65. This suggests that Sirt1 expression could potentially be regulated by NFκB.

### NFκB regulates the *SIRT1* promoter

**Figure 1:**
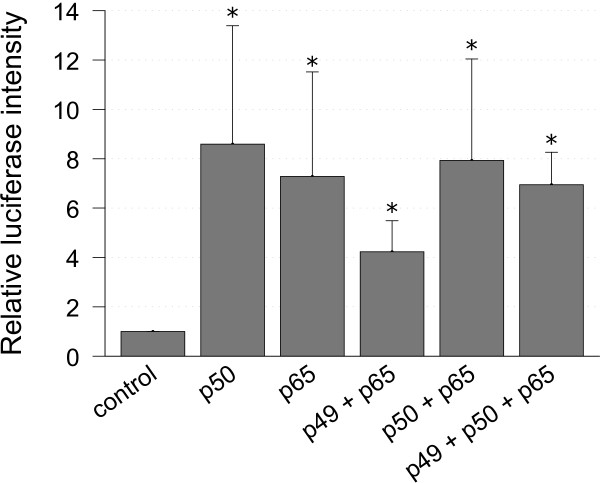
***SIRT1*-promoter activation after transfection of NFκB subunits.** Reporter plasmids containing the *SIRT1* promoter were cotransfected with expression vectors for different NFκB subunits in HEK-293 T cells. Luciferase reporter gene assay was done after 24 hours and protein concentration of cell extracts was used to normalize the results. To calculate relative values constitutive luciferase intensity (named control) was measured after transfection of the *SIRT1* reporter plasmid without NFκB expression plasmids. n = 3. *P ≤0.05 versus control group.

In order to further investigate whether NFκB was involved in the regulation of *SIRT1* at the promoter level, a luciferase reporter plasmid was generated, which contained a 2,228-bp-long fragment of the *SIRT1* promoter sequence. This construct was cotransfected in HEK293T cells together with different combinations of expression vectors of the NFκB subunits p49, p50 or p65. Luciferase activity of cell extracts was measured 24 hours after transfection and normalized to the protein content of the cell extracts. Figure 1 plots the results of three independent measurements of luciferase activity relative to the constitutive activity. The constitutive activity was determined by transfection of the reporter plasmid without cotransfection of any expression plasmids for NFκB. These results show clearly that the *Sirt1* promoter is activated in general by overexpression of NFκB. Statistical *P* values indicated in the diagram were calculated using a Mann-Whitney *U* Test and show significance for the difference of each NFκB subunit combination versus control (*P* ≤0.05). The different combinations of NFκB subunits we transfected in order to determine the effects of the different homo- and heterodimers of NFκB did not show significant differences among each other (*P* >0.1).

Our experiments showed overexpression of NFκB p50 subunit or NFκB p65 subunit alone to increase *SIRT1* promoter activity by an average of 8.6-foldand 7.3-fold. Overexpression of the two NFκB subunits p50 and p65 in combination was identified to have an activating effect on the *SIRT1* promoter with an average of about 7.9-fold, which is very similar to the effects of overexpression of the single subunits. The heterodimer p50/p65 represents the most common and best examined variant of NFκB, which makes these results the most interesting. The overexpression of p49 together with p65 also showed *SIRT1* promoter activation. The activating effect of this combination seemed to be lower with an average of only 4.2-fold, but the difference to the other subunit combinations is not significant. Overexpression of all three subunits together goes along with a seven-fold activation of the *SIRT1* promoter, which is similar to the other results. Transfection of the empty reporter plasmid (without *Sirt1* promoter) was used as negative control and did not show any luciferase activity.

### NFκB regulates *SIRT1* expression

**Figure 2:**
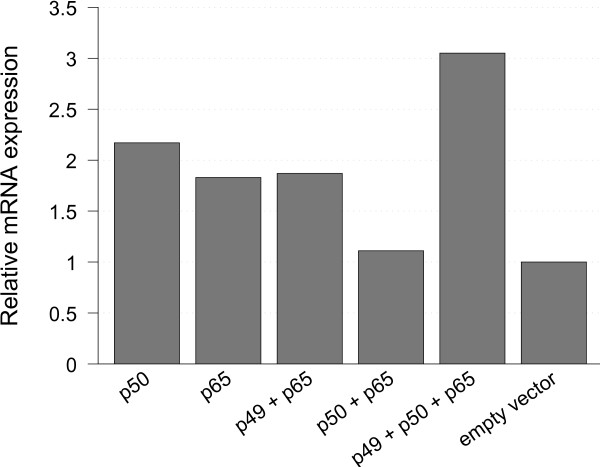
***SIRT1 *expression on mRNA level.** HEK-293 T cells were transfected with expression plasmids of different NFkB subunits and harvested 24 hours later. Cells transfected with empty vector were used as reference for calculation of relative values. The mRNA levels of Sirt1 gene were measured by real-time-PCR.

In order to examine whether the effects of promoter activation through overexpression of NFκB in fact had influence on the *SIRT1* expression levels, RNA from HEK293T cells was extracted after transient transfection with the NFκB expression constructs. In quantitative real-time PCR we observed a 1.8 to 3-fold upregulation for *SIRT1* at the mRNA level (Figure 2) in response to NFκB overexpression for nearly all combinations of NFκB subunits except in the combination of p50 with p65, which had only minor effects on *SIRT1* mRNA expression levels in this experiment. The little effect of the overexpression of p50 in combination with p65 on the mRNA level, does not fit with the remarkable promoter activating effects seen for this combination in the luciferase assay. A possible explanation may be based on the fact that the p50/p65 dimer is the most common dimer of NFκB, which has numerous physiological functions in the cell. Therefore, other regulating mechanisms may also be activated that could affect *SIRT1* expression.

### Functional assay of SIRT1 activity

**Figure 3:**
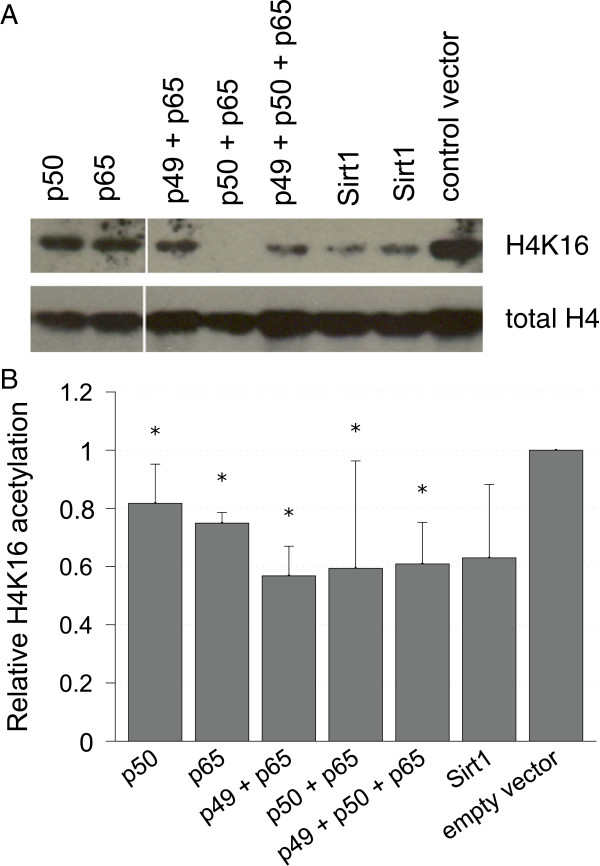
**Histone acetylation status in response to SIRT1 activity.** Cells were transfected with expression vectors for different NFκB subunits. Expression vector for *SIRT1* was used as a positive control. Histones were isolated after 24 hours and acetylation status was assessed by western blot. Transfection with empty vector was used as a reference control. **(A)** Detection was carried out with an antibody specific for acetylated histone H4_K16_ and with another antibody binding to histone H4 independently of the acetylation status (total H4). **(B)** Detection films of blots were scanned and quantified *in silico*. Values of H4_K16_ were normalized to total H4 and fold changes relative to control were calculated. n = 3. *P >0.05 versus empty vector.

A functional assay was carried out not only to investigate the levels of SIRT1 expression but also the effects of NFκB on cell metabolism and other proteins in association with SIRT1. In order to prove relevant changes in SIRT1 activity after NFκB overexpression we investigated the acetylation status of histone H4 lysine residue 16 (H4_K16_), which is targeted and deacetylated by SIRT1. We isolated the histone fraction of cells after transfection with defined NFκB subunits and analyzed the histone H4 acetylation status by western blot technique. Figure 3A shows a remarkable decrease of acetylated H4_K16_ in cells overexpressing NFκB. For quantification of the results, blots were scanned and acetylated histone H4 was normalized to the amount of total histone H4 on the same blot. To be sure, that the effects are not due to the circumstances of cell culture or transfection in general, which may change acetylation status of histones because it induces lots of metabolical reactions and stress, we compared the results of cells transfected with NFκB vector with those transfected with an empty vector. Figure 3B shows that the overexpression of NFκB subunits in all combinations resulted in a significant reduction of the relative amount of acetylated histone H4. The amount of reduction in cells overexpressing two or more NFκB subunits was similar to the reduction caused by direct overexpression of SIRT1. In cells overexpressing only one subunit of NFκB protein the effects are less but still detectable. These results suggest that overexpression of NFκB has activating effects on the deacetylation of histone H4, which could be mediated through induction of SIRT1 expression, particularly when two or three of the NFκB subunits are overexpressed, thus allowing for a formation of heterodimers.

### Binding of NFκB to *SIRT1* promoter

**Figure 4:**
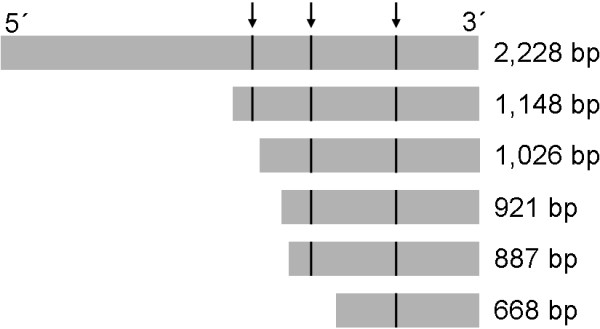
**Sirt1 promoter truncations.** Schematic illustration of Sirt1 promoter fragments used for electrophoretic mobility shift assay. The positions of NFkB binding sequences are marked by arrows.

To investigate the role of different putative NFκB binding sites, truncations of the *SIRT1* promoter were generated (Figure 4). The 2,228 bp and 1,148 bp *SIRT1* promoter fragments contained all three putative NFκB binding sites; fragments with length ranging from 1,026 bp, to 921 bp and 887 bp in length contained only two NFκB binding sites and the 668 bp fragment contained only one NFκB binding site.

**Figure 5:**
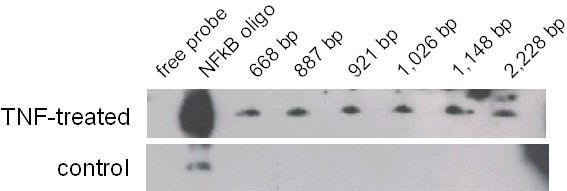
**Binding of NFκB to DNA fragments of truncated *SIRT1 *promoter.** U937 cells were treated with TNFα to induce NFκB expression. Nuclear extracts of TNFα-treated and untreated control cells were incubated with different biotin labeled DNA fragments of the *SIRT1* promoter and were analyzed by electrophoretic mobility shift assay. An NFκΒ oligonucleotide was used as the positive control.

An electrophoretic mobility shift assay was done to measure the binding of NFκB to the promoter and to investigate the relation of the three potential binding sites. In order to induce NFκB expression, cells were treated with TNFα. Nuclear extracts from both treated and untreated cells were isolated and incubated with biotin labeled DNA fragments. The reaction mixture was resolved on a 6% non-denaturing polyacrylamide gel. For all of the tested promoter fragments signals were detected (Figure 5), which proves binding of NFκB. The putative binding site contained in the short fragment is likely to be involved in the NFκB binding of the promoter, but because all of the fragments showed a signal, it is not possible to decide whether the binding is mediated solely by this single site or whether all three sites are involved. Nuclear extracts from untreated cells were used as negative controls. NFκB oligonucleotides were used as a positive control to prove induction of NFκB expression by TNFα and to compare the position of the bands on the blot. The positive control showed a signal that was much stronger than the labeled promoter fragments. This may be caused by the high sensitivity of the control, which also could explain the signal seen for the NFκB oligonucleotides when incubated with untreated cells.

## Conclusions

NFκB is a transcription factor that can be found in nearly all cell types. NFκB regulates immune reactions and is involved in the regulation of cellular stress resistance and apoptosis. Also, it has recently been shown that the NFκB binding sequence is a gene regulatory motif that is most essentially involved in the regulation of cellular aging. SIRT1 is a highly conserved protein involved in many cellular processes in both health and disease. It has been reported to be involved in the extension of cellular life span under conditions of caloric restriction in many species. Its positive influence on stress resistance of cells is widely known and its activation prevents apoptosis and cell cycle arrest. Since both proteins, SIRT1 as well as NFκB, are involved in the regulation of immune response, cellular stress, and aging, a regulatory connection between them would not really be very surprising. In some studies SIRT1 has been shown to interact with NFκB and to repress its activity by deacetylation [22,24]. But at present little information is available on the molecular mechanisms regulating the expression of *SIRT1*at the promoter level. In the study presented herein, we show that in addition to the already known regulation of NFκB by SIRT1, NFκB overexpression enhances SIRT1 promoter activity. Increased SIRT1 enzymatic activity caused by NFκB overexpression was shown in a functional assay. The direct binding of the transcription factor NFκB to the Sirt1 promoter was demonstrated by electrophoretic mobility shift assays. With these results a reciprocal feedback regulation of SIRT1 at the promoter level through transcription factor NFκB is shown to take place.

The conserved NFκB binding motif can be found three times within the human *SIRT1* promoter sequence. Distinguishing which of the three potential NFκB binding sites was responsible for the activation of the promoter could not be made since all of the created *SIRT1* promoter truncations showed binding of NFκB. With reporter plasmids containing the human *SIRT1* promoter sequence and luciferase assays, we were able to show that high expression levels of NFκB subunits were able to specifically activate *SIRT1* at the promoter level. Our experiments show that the NFκB binding motifs within the *SIRT1* promoter are functional, and that NFκB can act as a positive regulator of SIRT1 at the promoter level. In order to further examine whether NFκB overexpression on *SIRT1* promoter activation caused a real increase in SIRT1 expression, we performed RT-PCR experiments using cells overexpressing NFκB that showed elevated levels of *SIRT1* mRNA for most of the used NFκB subunits. As a functional assay to measure SIRT1 activity the acetylation status of histone H4_K16_, which is one of the most common targets of the deacetylase SIRT1, was examined. The decrease in acetylation of H4_K16_ under conditions of NFκB overexpression indicates not only that SIRT1 protein levels are increased, but also that this has measurable effects on downstream targets. This means that at least some of the signaling cascades and pathways that activate NFκB may influence the expression of SIRT1 and the acetylation status of its target proteins. The involvement of both of these proteins in the development and progression of numerous diseases such as cancer, arteriosclerosis or diabetes renders this regulation mechanism an interesting target for further investigations with regard to the development of novel molecular therapeutics affecting SIRT1 regulation. SIRT1 is one of the most interesting targets for potential epigenetic therapeutics and the ongoing attempts to create specific activators or deactivators for SIRT1 may benefit from better knowledge about its regulation by intercellular transcription factors. Taken together, these findings provide new insights into the regulation of SIRT1 and interactions between NFκB and SIRT1. They may promote a better understanding of the mechanism involved in regulation of proteins that control procedures as important as metabolism and aging.

## Methods

### Antibodies and plasmids

Anti-histone H4 monoclonal antibody (anti-H4) was purchased from Abcam (Cambridge, UK) and antibody specific for Histone H4 acetylated at lysine 16 (anti-H4_K16_) was purchased from Upstate via Biomol, (Hamburg, Germany). Secondary antibodies (anti-rabbit and anti-mouse horseradish peroxidase conjugates) were purchased from Santa Cruz Biotechnology (Santa Cruz, California, USA).

NFκB expression plasmids for subunits p49, p50 and p65 were based on pRSV expression vector systems and were provided by the National Institutes of Health (NIH) AIDS Research and Reference Reagent Program. For construction of luciferase reporter plasmids, human *SIRT1* promoter from genomic DNA was amplified by PCR. The amplified region spanned the entire *SIRT1* promoter region. The promoter was inserted into the luciferase reporter plasmid pGL2-basic, which was purchased from Promega (Mannheim, Germany). All plasmids were propagated using competent DH5α E. coli and were isolated with a Nucleobond PC100 Kit from Macherey & Nagel (Düren, Germany). All transfection experiments were carried out in HEK293T cells.

### Luciferase reporter gene assays

HEK293T-cells were cultured in 12-well plates with DMEM supplemented with 10% FCS and were cotransfected with the luciferase *SIRT1* promoter or empty pGL2basic vector constructs and different combinations of expression vectors encoding NFκB subunits. In order to normalize the total amount of DNA that was used per transfection, empty vector was added in order to adjust the total amount of DNA. Transfection was done using the nanoparticle-based transfection reagent Nanofectin (PAA, Pasching, Austria) and medium was changed 2 hours after transfection. Transfection efficiency was assessed by UV-microscopy after transfection of green lantern constructs (Promega, Mannheim, Germany). Cells were harvested after 24 hours and luciferase activity was measured using the luciferase reporter assay system (Promega, Mannheim, Germany) in accordance with the manufacturer’s instructions. The activity of each sample was normalized to its protein concentration measured on a UV-Vis spectrophotometer Nanodrop™ ND1000 (PEQLAB, Erlangen, Germany) using DC Protein assay (BioRad, Hercules, California, USA) based on the Lowry method. Relative activity was calculated as fold difference in relation to the control vector pGL2basic. Luciferase assays were performed in duplicates and every experiment was repeated at least twice.

### Quantitative real-time PCR

Total RNA from HEK293T cells was extracted with the Trifast reagent (Peqlab, Erlangen, Germany) in accordance with the manufacturer’s instructions. Total RNA was treated with Turbo DNAse (Life Technologies, Carlsbad, USA) and was reverse transcribed with the High capacity cDNA Reverse Transcription Kit from Applied Biosystems (Darmstadt, Germany). The cDNA samples were amplified in quantitative real-time PCR using a Light Cycler 2.0 system and DNA Master SYBR Green Kit both from Roche (Penzberg, Germany). The primers that were used for amplification of human *SIRT1* were as follows:

forward primer 5′- ACGCTGGAACAGGTTGCGGGA - 3′,

reverse primer 5′- AAGCGGTTCATCAGCTGGGCAC - 3′.

Thermal reaction cycles were as follows: initial cycle at 95°C for 10 min, which was then followed by 45 cycles at 95°C for 10 sec, 60°C for 4 sec and 72°C for 16 sec. GAPDH was used as an internal control to normalize measured Ct values of each sample, and data were analyzed using the delta-delta Ct method.

### Extraction of histones and western blot

HEK293T cells were cultured in 12-well plates and were harvested with 100 μl lysis buffer after incubation for 24 hours. While cells were being mixed vigorously, 25 μl of 2 M sulphuric acid were added drop by drop, and cells were then placed on ice for 1 h. After centrifugation at 13,000 rpm, supernatants were collected, mixed with 400 μl of 20% trichloroacetic acid and incubated on ice for histone precipitation. After 1 h the mixture was spun down and the pellets containing histones were washed twice with acidified acetone (0.1% HCl) and once with pure acetone prior to resuspension in water. Extracted histones were separated on a 15% polyacrylamide gel and blotted onto a nitrocellulose membrane according to the full-wet western blot technique. Acetylated histones were detected with anti H4_K16_ antibody. For detection, the ECL plus western blotting detection reagent from GE Healthcare (Little Chalfont, UK) was used. After that, an antibody binding to H4 independent of the acetylation status was used in order to detect total histone H4. Blots were scanned and quantifications were calculated with the ImageQuant5.1 software (GE Healthcare, Little Chalfont, UK). Signal of acetylated H4 was normalized to the signal of total H4 in order to eliminate effects depending on the western blot technique itself.

### Promoter truncation and electrophoretic mobility shift assay (EMSA)

The generation of the different promoter fragments was done with different methods. The fragment with 921 bp was generated by exonuclease digestion. Fragments with 1,148 bp and 1,026 bp were generated through restriction enzyme digestion and subsequent re-ligation. Fragments with 887 bp and 668 bp were amplified by PCR from genomic DNA and inserted into an empty vector. Plasmids were transformed into *E. coli DH5a* for propagation and extracted accordingly. A total of 2 μg plasmid DNA were digested with restriction enzymes (Table 1) and then run on a 0.8% agarose gel. Digested fragments from each construct were cut out while the gel was monitored under UV control, and DNA elution was done with a Silica Beads DNA gel extraction kit (Fermentas, Waltham, USA). DNA from each construct was labeled with biotin using the Biotin 3′ End DNA Labeling Kit (Pierce Biotechnology, Waltham, USA). In order to stimulate NFκB expression, U937 cells were treated with recombinant TNFα at a concentration of 10 ng/ml for 30 minutes. Control cells were left untreated. Nuclear extracts (10 μg) were then incubated with a specific biotin-labeled DNA sample before electrophoretic mobility shift assay (EMSA) was done using a Light Shift Chemiluminescent EMSA kit (Pierce Biotechnology, Waltham, USA) in accordance with the manufacturer’s instructions.

**Table 1:** **Enzymes for digestion of *SIRT1 *promoter truncations**

**Fragment length**	**Enzymes for plasmid digestion**
2,228 bp	*Hind3*
1,148 bp	Bgl2 and *Hind3*
1,026 bp	*Kpn1* and *Hind3*
921 bp	Sma1
887 bp	*Bgl2* and *Hind3*
668 bp	*Bgl2* and *Hind3*

## Abbreviations

NFκB: Nuclear factor of kappa light polypeptide gene enhancer in B-cells; SIRT1: Sirtuin 1.

## Competing interests

The authors declare that they have no competing interests.

## Authors’ contributions

UM, JK, NE, and WA conceived and designed the experiments. JK, NE, and WA analyzed the data. JK wrote the first draft of the manuscript. UM contributed to the writing of the manuscript. UM GH, WA, and NE made critical revisions. All authors reviewed and approved the final manuscript.
